# *In Vitro* Analysis of Fibronectin-Modified Titanium Surfaces

**DOI:** 10.1371/journal.pone.0146219

**Published:** 2016-01-05

**Authors:** Yu-Chi Chang, Wei-Fang Lee, Sheng-Wei Feng, Haw-Ming Huang, Che-Tong Lin, Nai-Chia Teng, Wei Jen Chang

**Affiliations:** 1 School of Dentistry, College of Oral Medicine, Taipei Medical University, Taipei, Taiwan, ROC; 2 School of Dental Technology, College of Oral Medicine, Taipei Medical University, Taipei, Taiwan, ROC; 3 Graduate Institute of Biomedical Materials & Tissue Engineering, College of Oral Medicine, Taipei Medical University, Taipei, Taiwan, ROC; 4 Dental Department, Taipei Medical University Hospital, Taipei, Taiwan, ROC; 5 Dental Department of Taipei Medical University, Shuang-Ho Hospital, Taipei, Taiwan, ROC; University Paul Sabatier, FRANCE

## Abstract

**Background:**

Glow discharge plasma (GDP) procedure is an effective method for grafting various proteins, including albumin, type I collagen, and fibronectin, onto a titanium surface. However, the behavior and impact of titanium (Ti) surface modification is yet to be unraveled.

**Purpose:**

The purpose of this study is to evaluate and analyze the biological properties of fibronectin-grafted Ti surfaces treated by GDP.

**Materials and Methods:**

Grade II Ti discs were initially cleaned and autoclaved to obtain original specimens. Subsequently, the specimens were GDP treated and grafted with fibronectin to form Ar-GDP (Argon GDP treatment only) and GDP-fib (fibronectin coating following GDP treatment) groups. Blood coagulation test and MG-63 cell culture were performed to evaluate the biological effects on the specimen.

**Results:**

There was no significant difference between Ar-GDP and GDP-fib groups in blood compatibility analysis. While in the MTT test, cellular proliferation was benefited from the presence of fibronectin coating. The numbers of cells on Ar-GDP and GDP-fib specimens were greater than those in the original specimens after 24 h of culturing.

**Conclusions:**

GDP treatment combined with fibronectin grafting favored MG-63 cell adhesion, migration, and proliferation on titanium surfaces, which could be attributed to the improved surface properties.

## Introduction

Osseointegration [1], which refers to the process through which the mature living bone establishes a direct structural and functional connection with the implant without any intervening soft or fibrous tissue, is an important phenomenon favoring the effective fixation of implants to bone. Successful osseointegration depends on both the chemistry of the substrate and its surface texture. The surface roughness of titanium implants greatly affects the rate of osseointegration and biomechanical fixation.[2,3] In addition, cell adhesion and spreading are important parameters influencing the implant engineering.

Cellular responses in the vicinity of the implants are strongly affected by surface hydrophilicity, roughness, texture, chemical composition, charge and morphology.[4,5] Given this fact, several studies have attempted to enhance the adhesion and activity of osteoblastic cells on the surfaces by depositing functional proteins onto the titanium surface. [6,7] Of the different methods, glow discharge plasma (GDP) procedure is a popular method for realizing surface modification of biomaterials. Several studies have demonstrated the advantages of GDP method in sterilizing and modifying the surface of the biomaterials [8–10]. The XPS results showed that the Ti surface after plasma treatment was leaving chemistry uncharged.[11] Most importantly, this method offers the possibility of forming bio-functional groups and functional proteins on the titanium surfaces [12,13]. Given all these advantages, this method can be considered as an efficient tool for improving the biocompatibility of biomaterials.

In general, contact between the implants and bone can be improved by adopting surface treatment methods, such as sand blasting, sol-gel dip coating, titanium nitride oxide (TiNOx) coating, plasma spraying, acid etching, and various combinations thereof.[14–16] Furthermore, the biocompatibility of titanium surface could be improved by grafting several functional proteins, such as extracellular matrix proteins (ECM), type I collagen, and albumin using the GDP method.[12,17,18] Bioactive adhesive proteins, such as the extracellular matrix protein fibronectin, have been used to facilitate the surface properties of titanium. The titanium surface with fibronectin coating following GDP treatment were more hydrophilic and had greater surface roughness. Meanwhile, the FITC labeling showed that the number of fibronectin dots on the titanium surface increased positively with the concentration of fibronectin solution used.[19] The attachment of cells through fibronectin binding were measured.[20] The attachment and differentiation of osteoblast-like cells were enhanced on Ti6Al4V titanium surface through fibronectin coating.[21–24] However, the cell culture study on commercially pure titanium (cpTi) remains an important focus of investigation.

Fibronectin is known to play an important role in cell attachment, growth, migration and differentiation. In addition, it is also reported to participate in platelets adhesion and aggregation via the integrin αIIbβ3 (glycoprotein IIb/IIIa) receptors on platelet membrane.[25] However, with the advance of technology, the hemolysis rate and blood coagulation time could be lower and prolonged on titanium surface after fibronectin coating.[26] The blood compatibility of titanium surface with fibronectin coating after GDP treatment has not been studied yet.

To this end, the present study aims to evaluate the biological properties of fibronectin-grafted titanium surface prepared by GDP method. In the process, GDP was used to create bio-functional amine groups on titanium surfaces. Subsequently, fibronectin was grafted onto the amine groups with glutaraldehyde (GA) cross linker. Blood compatibility analysis and cell culture experiments using osteoblast-like cells MG-63 were used to evaluate the bio-functionality of the modified titanium surface.

## Materials and Methods

### Preparation of titanium surface

10 mm diameter Grade II titanium discs (BioTech One Inc., Taipei, Taiwan) were used in this study. The preparation method was clearly described previously.[18,19] The samples were cleaned in an ultrasound bath with detergent, acetone, and distilled water for 15 min separately to remove the surface contaminants. Subsequently, the cleaned titanium discs were autoclaved at 121°C for 20 min and dried at 40°C in a conventional oven. The titanium discs thus obtained are henceforth referred to as “original specimens.”

### Glow discharge plasma cleaning and protein grafting

**Fig 1:**
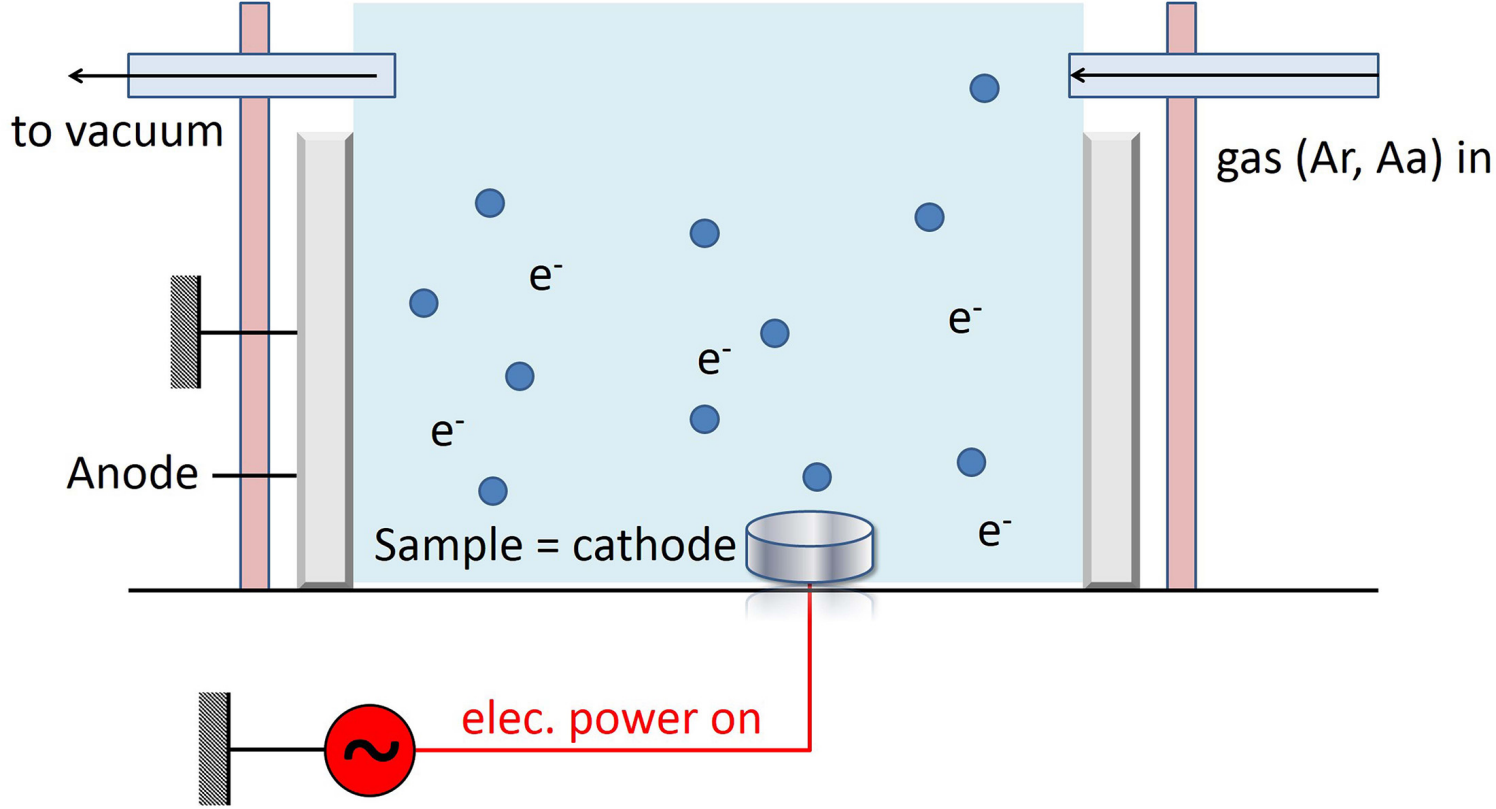
Schematic of the flow discharge plasma system. The blue circles represented the gas ions (argon or allylamine) which were used to modify the titanium surface.

**Fig 2:**
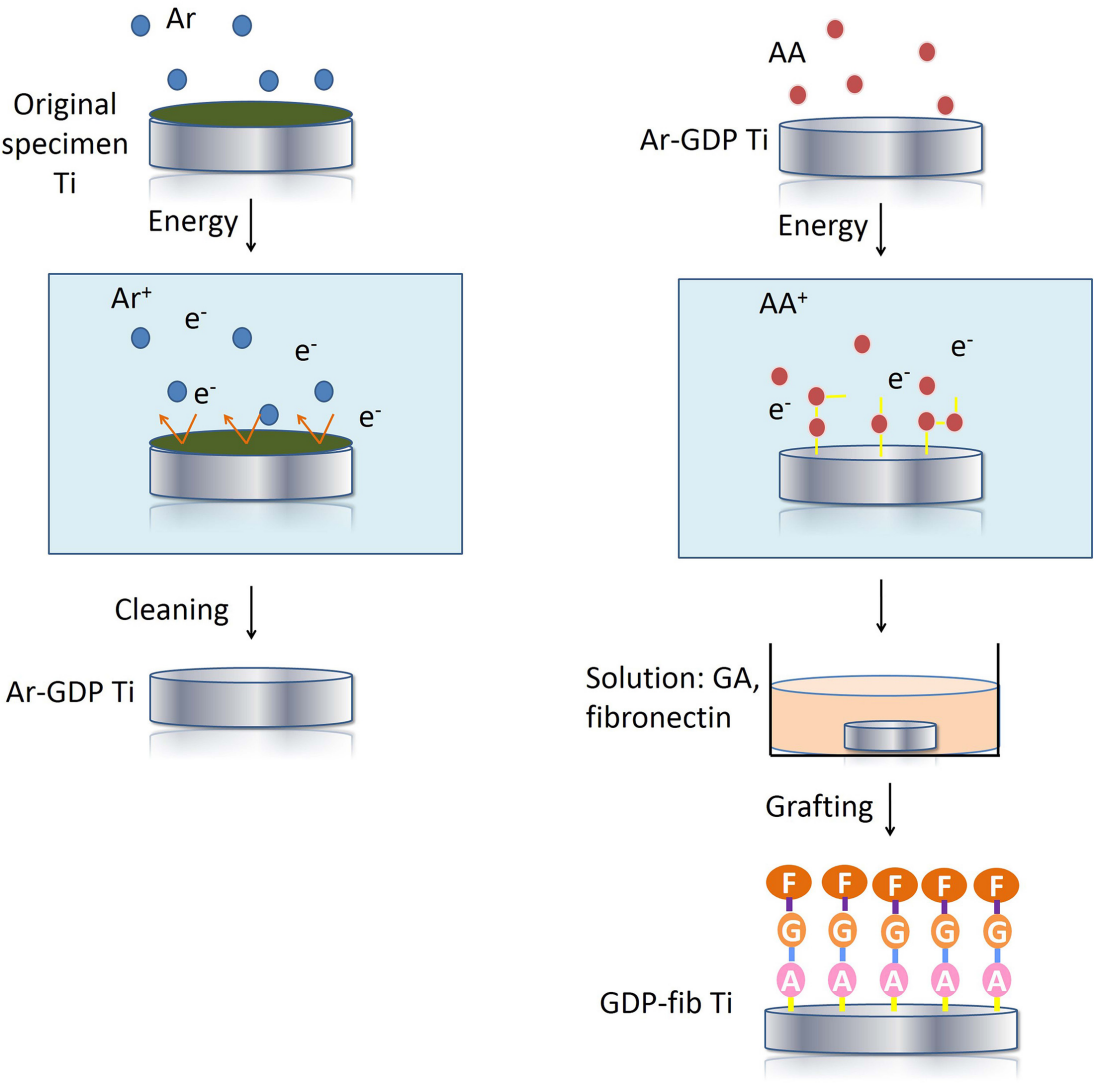
Schematic of sample preparation. (a)The surfaces of Ti discs (original specimens) were cleaned with argon-based GDP. These titanium discs were then labeled as “Ar-GDP.” (b) Amine groups were grafted onto the Ar-GDP surfaces in the GDP reactor fed with allylamine (AA) gas. These specimens were subsequently treated in glutaraldehyde (GA) and fibronectin solutions and were labeled as “GDP-fib”.

The original specimens were cleaned by GDP (PJ; AST Products Inc., North Bellericca, MA, USA) with argon. This process involves GDP treatment under argon gas at room temperature under the following conditions: power, 85 W; frequency, 13.56 MHz; pressure, 100 mtorr for 15 min (Fig 1).[19] The titanium discs thus obtained are referred to as “Ar-GDP” (Fig 2a).

Subsequently, NH groups were adhered onto the titanium surfaces in the GDP reactor fed with allylamine (AA) gas at room temperature under the same conditions as mentioned above except treatment time was increased to 30 min. Thereafter, the specimens were placed in a 3% glutaraldehyde solution (Merck, NJ, USA) for 30 min. Then, the titanium specimens were placed in a solution with 5 μg/ml fibronectin (Sigma-Aldrich Co., St. Louis, MO, USA) for 24 hours to form fibronectin coating on the surface of titanium discs. A tris-phosphate buffer (pH 7.4) was used to stop the chain reaction between amine group and fibronectin (immersed for 30 min).[19] The titanium discs thus obtained are referred to as “GDP-fib” (Fig 2b).

### Surface topography evaluation

The surface characteristics of Ar-GDP and GDP-fib were examined with scanning electron microscope (SEM) (Model 2400; Hitachi, Ltd) in order to evaluate the topography of Ti surface after fibronectin coating.

### Blood coagulation test

Eight 5 month old New Zealand white rabbits of mean weight 3 kg were used for the analysis. All procedures were approved by the Animal Care and Ethics Committee of Taipei Medical University, Taipei, Taiwan (affidavit of approval No. LAC-2013-0265) (S1 File). The study was conducted according to the principles of the 2010 Basel Declaration. This animal research was conducted in accordance to the ARRIVE (Animal Research: Reporting of *In Vivo* Experiments) guidelines developed in NC3Rs (National Centre for the Replacement, Refinement and Reduction of Animal Research) (S2 File).[27]

The coagulation of the blood on the surface of blank Petri dish and Ar-GDP and GDP-fib titanium disks were evaluated by using spectrophotometry. Fresh rabbit blood was used for each experiment. During the test, 10 ml of blood from each rabbit was drawn with a syringe with 24G needle (TERUMO Co., Tokyo, Japan) from the marginal vein on the right or left ear under animal fixation with a fixation box. The rabbits were alive and kept for further study after the blood collection. Subsequently, a 200 μl of fresh rabbit blood was placed on the surface of the empty Petri dish and 24-well Petri dishes (Nunclon; Nunc, Roskilde, Denmark) with the titanium samples. After 10, 20, 30 and 40 min, 2 ml of distilled water was placed in the wells and the Petri dishes were subsequently incubated at 37°C for 10 min to disperse the free RBCs. The absorbance values of the wash solutions were colorimetrically measured by using an ELISA reader (Model 2020, Anthos Labtec Instruments, Eugendorf, Wals, Austria) at 540 nm. Each absorbance value represented the average of six measurements.

Blood coagulation of Ar-GDP and GDP-fib specimens were expressed as a percentage and compared to that of the blank plates (100%). The remainders of Ar-GDP and GDP-fib were used for statistical analysis. High absorbance value, which is also demonstrated as the lower absorbance value remainder, implies a low amount of blood coagulation.

### Cell vitality test

In the process, MG-63 (ATCC CRL-1427) osteoblast-like cells, at a concentration of 1 × 10^4^ cells/ml, were seeded onto the surface of the original specimen, Ar-GDP and GDP-fib titanium discs in the 24-well Petri dishes (Nunclon; Nunc, Roskilde, Denmark). During all experiments, the cells were initially incubated without medium for 10 hours to ensure attachment. The cells were then cultured in Dulbecco’s modified Eagle’s medium (DMEM; HyClone, Logan, UT) supplemented with L-glutamine (4 mmol/L) with 10% fetal bovine serum (FBS), and 1% penicillin streptomycin. Cultures were incubated in 5% CO_2_ atmosphere at 37°C and 100% humidity. Accordingly, the time, when DMEM was added, was defined as the 0^th^ hour (T0) for every test.[17]

At 0^th^ (T0) and 24^th^ (T24) hour of culture, the culture media were removed and the discs were rinsed three times with PBS. The samples were then fixed in 2.5% GA and 2% paraformaldehyde solution for 30 min. Subsequently, for post-fixation, the samples were rinsed in 1% osmium tetroxide for 1 hour. The titanium discs were then dehydrated in 70%, 80%, 90%, 95%, and 100% series ethanol and dried in a critical point dryer (HCP-2; Hitachi Ltd, Tokyo, Japan). Thereafter, a thin layer of palladium gold was coated on the samples with a sputter coater (IB-2; Hitachi, Ltd, Tokyo, Japan). The morphological features of the cells were examined by using a Hitachi S-2400 electron microscope (Model 2400; Hitachi, Ltd). Two samples were prepared for each group.

Cell vitality was determined using an MTT ((3–4,5-dimethylethiazol-2-yl)-2,5-diphenyl tetrazolium bromide) reduction assay. Tetrazolium salt (MTT kit, Roche Applied Science, Mannheim, Germany) was added and metabolically reduced to colored formazan by mitochondrial dehydrogenase in viable cells, as indicated in the manufacturer’s instructions. After solubilizing, the formazan dye was added with 500 μl of dimethyl sulfoxide for 5 min. Subsequently, the optical density of the medium was determined by using the ELISA reader at 570 nm. Optical density results were expressed as a percentage of that of the original specimen. In this study, four samples were prepared for each group.

### Statistical analysis

Biological characters of the modified titanium surfaces were evaluated by performing hemocompatibility and MTT assay. The morphology of the MG-63 cell cultured samples was observed by using SEM. For all assays, differences between tested groups were evaluated by Student’s *t*-test. Significance levels were set to *P* ≤ 0.05.

## Results

### Surface topography evaluation

**Fig 3:**
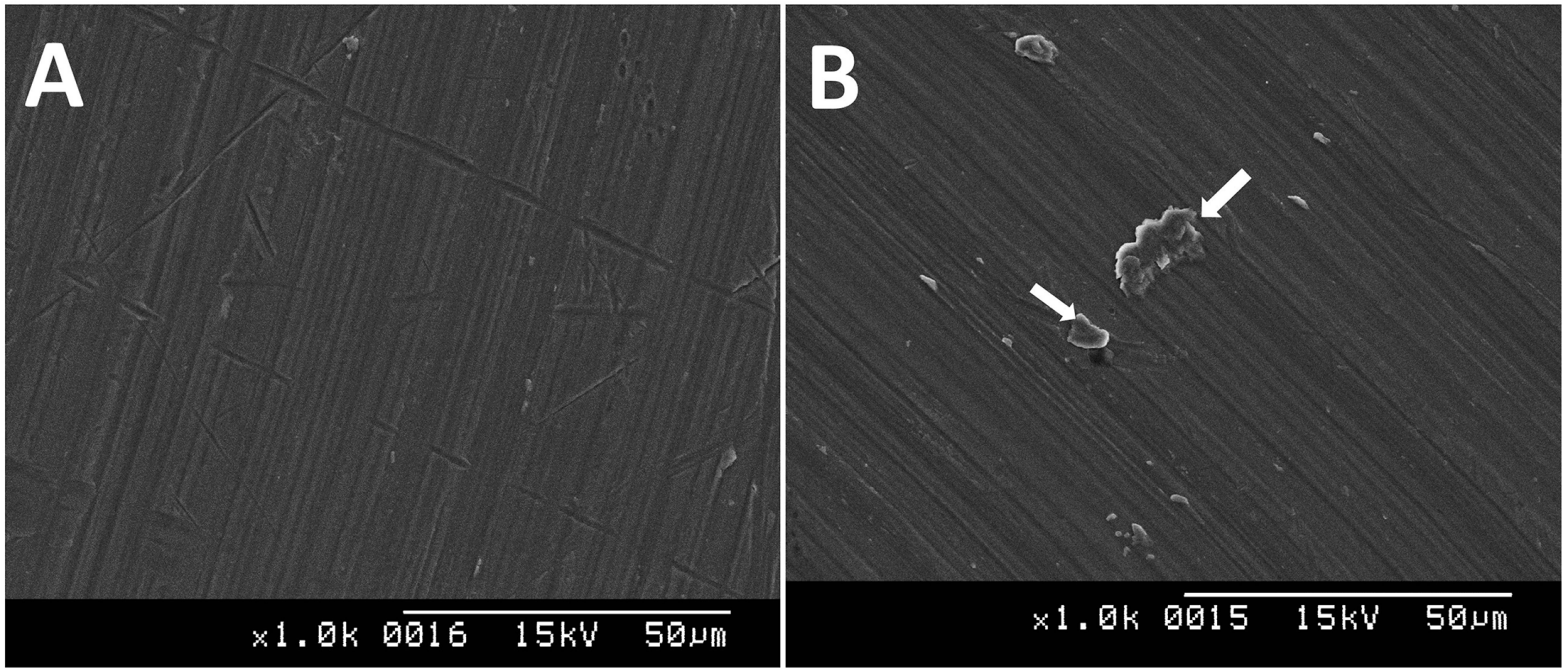
Scanning electron microscopy images of the Ar-GDP (a) and GDP-fib (b) titanium discs. Irregular folding of the fibronectin was revealed on the surface of fibronectin-grafted titanium disks *(white arrows)*.

The surface of Ar-GDP discs showed planar morphology with parallel grooves (Fig 3a). However, the GDP-fib samples had a homogenous layer with irregular folding structure of protein (Fig 3b).

### Blood coagulation test

**Fig 4:**
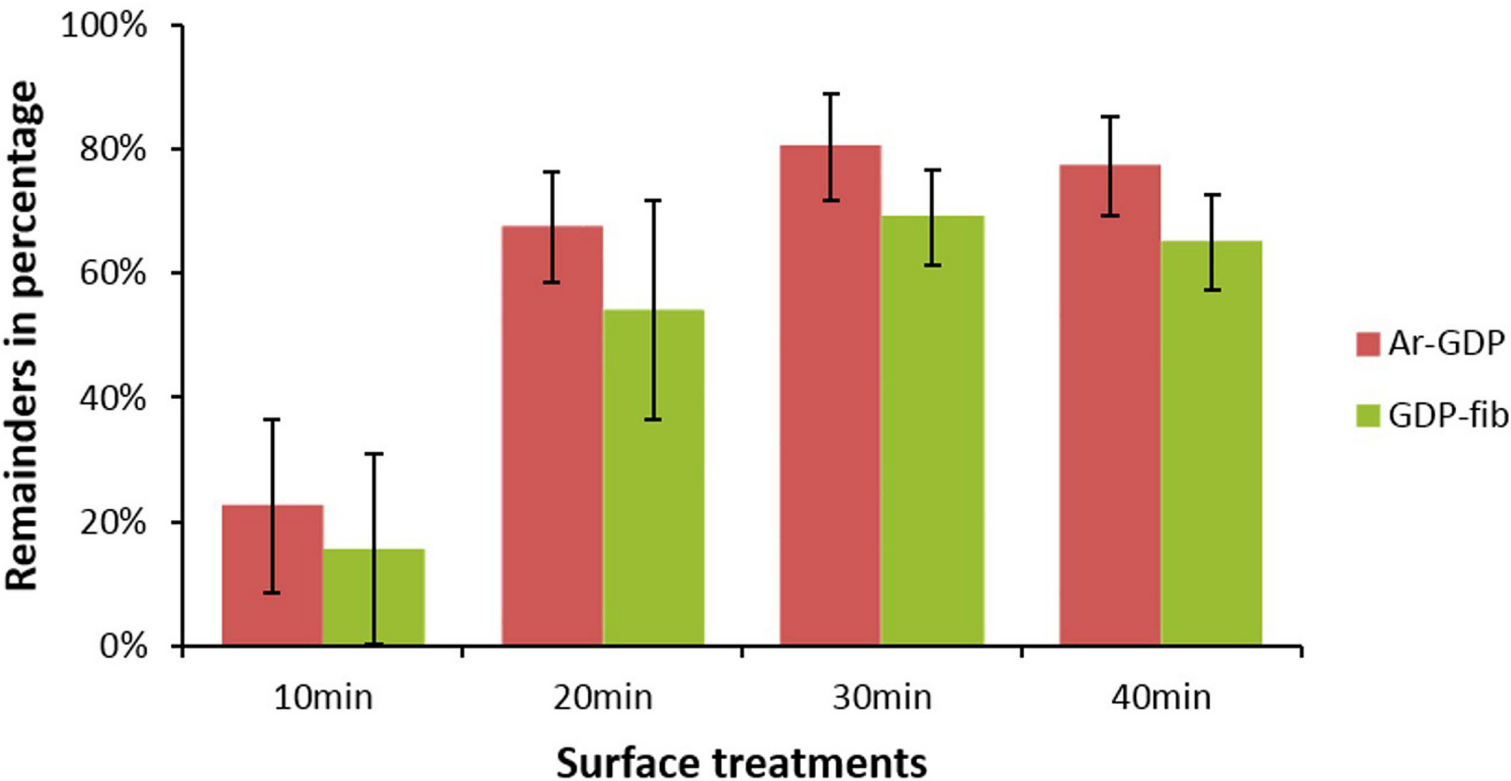
Blood coagulation of Ar-GDP and GDP-fib samples was expressed as percentage and compared to that of the blank plate (100%). There was no significant difference between two groups.

The results of blood coagulation test showed that the hemocompatibility of titanium discs decreased after fibronectin grafting; nevertheless, there was no significant difference between two groups (Fig 4). The hemocompatibility of Ar-GDP and GDP-fib samples were expressed as a percentage and compared to that of the blank plate (100%). The remainders of the Ar-GDP titanium discs were 22.6%, 67.5%, 80.3% and 77.3% at 10, 20, 30 and 40 min of blood coagulation, respectively. Similarly, in the case of GDP-fib discs, the remainders were 15.6%, 54.1%, 68.9% and 65.0% at 10, 20, 30 and 40 min of blood coagulation, respectively. As is seen, the remainder of the Ar-GDP titanium discs was not significantly greater than the GDP-fib discs at each set point of time.

### Cell vitality test

**Fig 5:**
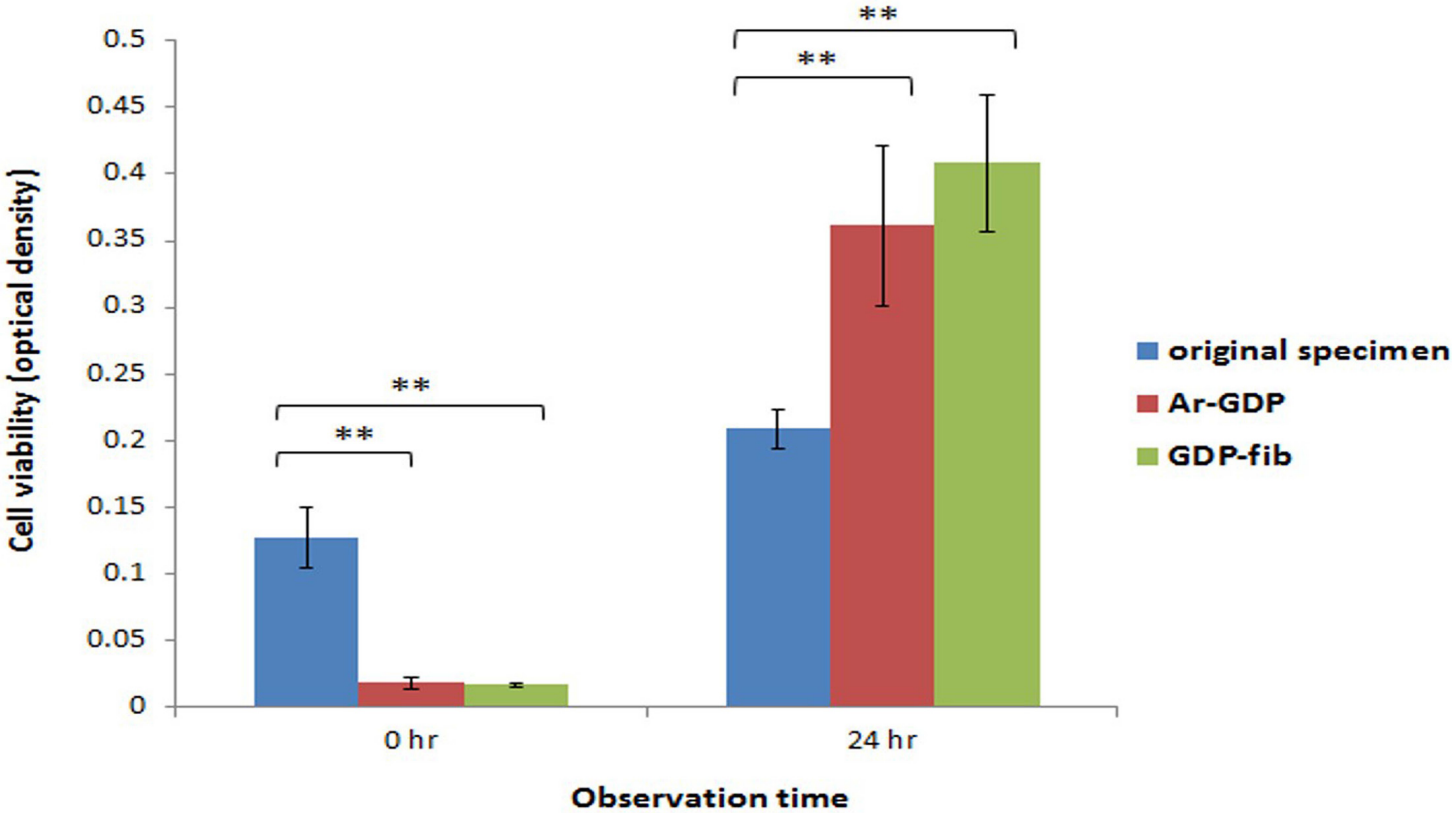
The MTT assay of MG-63 cells. Compared to the original specimen, both on the surface of GDP-treated and fibronectin-modified titanium, the MG-63 cells viability was significantly enhanced after 24 hours of culture (**P < 0.01).

According to the MTT assay analysis, the number of MG-63 cells on Ar-GDP and GDP-fib titanium specimens after 24 hours (T24) of culture was greater than that on the original specimens (Fig 5). Besides, the cells on the original specimens showed significantly less cell proliferation when compared to the cells on the Ar-GDP and GDP-fib titanium discs (*P* < 0.01). There was no significant difference between the Ar-GDP and GDP-fib groups.

### MG-63 cell morphology evaluation

**Fig 6:**
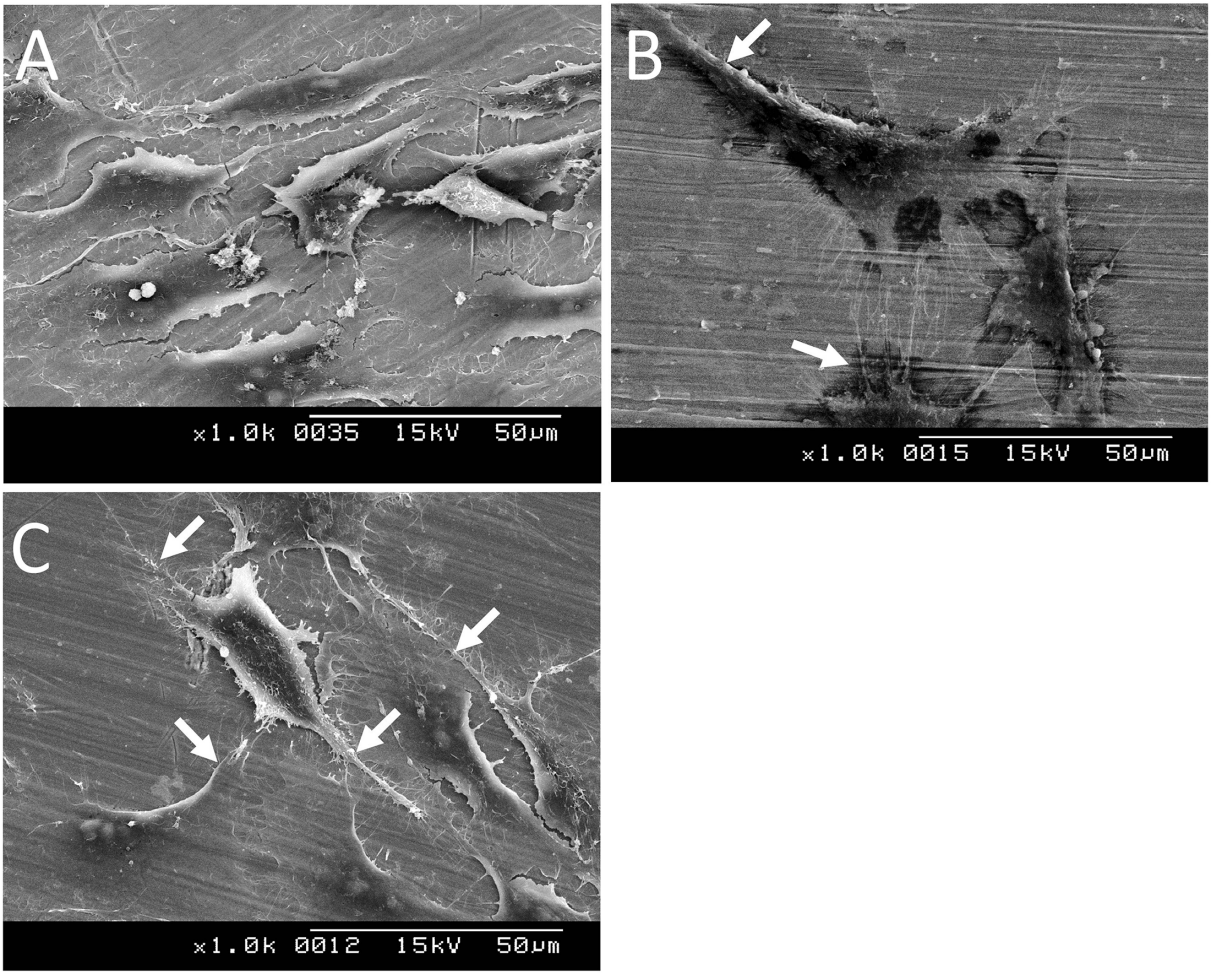
Scanning electron microscopy images of MG-63 cells cultured on original specimen (a), Ar-GDP (b), and GDP-fib (c) titanium discs after 24 h. Cells showed morphological alternation from spindle to more-stellar shapes, and extensive process (*white arrows*).

The MG-63 cells exhibited distinctly different morphologies on the SEM images of original specimens, Ar-GDP, and GDP-fib titanium surfaces. The MG-63 cells SEM images after 24 hours of culture (Fig 6) showed flaky shape cells seeded onto the original specimens (Fig 6a). The MG-63 cells were spread out in an even way and appeared to form a thin and continuous monolayer on the Ar-GDP titanium surfaces (Fig 6b). Nevertheless, morphological alternation of MG-63 cells, from spindle to more stellar shape with extensive filopodia contacting with each other and with the substrate (Fig 6c), could be observed when the fibronectin-grafted titanium discs were used as substrates.

## Discussion

The objective of this study was to gain in-depth insights of the biological effects, including hemocompatibility and cellular reaction, of the titanium surfaces after GDP and fibronectin modification. Therefore, we analyzed the surfaces prepared using different methods. The surface roughness and wettability of the modified titanium surfaces have already been reported in previous studies. In the study, fibronectin-grafted specimens had higher hydrophilicity and greater surface roughness than the GDP-treated specimens.[19] The FITC labeling ensured the formation of fibronectin coating. Grafted fibronectin on titanium surface was spotty distribution instead of monolayer formation. In addition, the number of fibronectin dots on the titanium surface increased positively with the concentration of fibronectin solution used.[19]

The normal wound healing process is divided into four stages, namely, hemostasis, inflammatory, proliferative and remodeling phases. Coagulation (thrombogenesis), the process by which blood forms clots, is an important part of hemostasis.[28] The GDP treatment could enhance the adhesion of blood cells onto the surface of the biomaterial, thereby favoring aggregation and blood coagulation on polymer-based biomaterials.[29] Because of the effect, the wound healing and osseointegration will be promoted. In order to improve hemocompatibility, various proteins, such as collagen, were adsorbed onto the titanium surfaces.[12,30,31] According to this study, the blood coagulation decreased on the surface of fibronectin-grafted titanium discs. In other words, there might be more blood cells in contact with the surface of the fibronectin-grafted discs and not trapped into the clot. This might have accelerated the differentiation. In addition, there is no significant difference between Ar-GDP and GDP-fib groups. Overall, the adhesion and aggregation of platelets were not significantly minimized by the presence of the thin fibronectin layer, but cellular proliferation and differentiation might have been benefited.

In general, MG-63 cells, a human osteoblast-like cell line, are being used to evaluate the biological effects of different implant surface designs. Despite proliferating more rapidly than the other human bone-derived cells, MG-63 cells perform many osteoblastic traits that are characteristic of bone-forming cells. MG-63 cells show enhanced alkaline phosphatase activity and actin-ring formation following 1α, 25-dihydeoxyvitamin D_3_ administration. This is again a typical characteristic of bone-derived cells.[31,32] Thus far, several *in vitro* studies with different cell culture analysis have shown that the osteoblast proliferation, differentiation, and matrix synthesis is greatly influenced by the nature of implant surface.[33,34] In general, cells cultured on rough surfaces tended to demonstrate more differentiated osteoblasts than those cultured on smoother surfaces. [35,36]

The results performed in the present study supported the abovementioned reports. The surfaces of GDP-fib titanium discs (0.400μm; 5.4 ± 0.8°) were significantly rougher and more hydrophilic than Ar-GDP specimens (0.170 μm; 36.8 ± 8.5°).[19] The MTT assay (Fig 5) and SEM images (Fig 6) demonstrated that the number of cells on Ar-GDP and GDP-fib specimens were greater than that on the original specimens after 24 hours of culture. The results of MTT assay showed that the cell number was greater on original specimen at T0; nevertheless, the number of MG-63 cells on Ar-GDP and GDP-fib titanium specimens was greater than that on the original specimens at T24. The result might imply that, during the first 10 hours of attachment without DMEM, MG-63 cells tended to proliferate on original specimens while tended to differentiate on other two groups due to the different contact surface. The hypothesis was supported by the results of SEM morphology evaluation. The result of T24 culture also suggested that the GDP treatment and fibronectin grafting could enhance the proliferation rate of MG-63 cells. Besides, the cell viability of GDP-fib group was more than that of Ar-GDP group. On the basis of these results, it is reasonable to conclude that the fibronectin-grafted titanium specimens that are treated by GDP method could reduce the osseointegration period and improve osseointegration in the early stages of dental implant fixation.

In conclusion, the results obtained in this study indicate that the blood coagulation was not significantly different with fibronectin coating after GDP pretreatment. However, the cell adhesion, migration, and proliferation were benefited from the presence of fibronectin grafting. This approach allows to find the biological modified implant surface and to examine the effects that could possibly occur during the material-cell interaction. More *in vitro* and *in vivo* studies are certainly necessary for the identification of future endosseous implant design.

## Supporting Information

S1 File LAC-2013-0265. The affidavit of approval from the Animal Care and Ethics Committee of Taipei Medical University, Taipei, Taiwan.
(PDF)

S2 File The ARRIVE checklist. (PDF)

## References

[1] Branemark P.I. Osseointegration and its experimental background. *J Prosthet Dent* 1983, 50, 399–410. 6352924 10.1016/s0022-3913(83)80101-2

[2] Wennerberg A.; Hallgren C.; Johansson C.; Danelli S. A histomorphometric evaluation of screw-shaped implants each prepared with two surface roughnesses. *Clin Oral Implants Res* 1998, 9, 11–19. 9590940 10.1034/j.1600-0501.1998.090102.x

[3] Lazzara R.J.; Testori T.; Trisi P.; Porter S.S.; Weinstein R.L. A human histologic analysis of osseotite and machined surfaces using implants with 2 opposing surfaces. *Int J Periodontics Restorative Dent* 1999, 19, 117–129. 10635177

[4] Oshida Y.; Hashem A.; Nishihara T.; Yapchulay M.V. Fractal dimension analysis of mandibular bones: Toward a morphological compatibility of implants. *Biomed Mater Eng* 1994, 4, 397–407. 8000293

[5] Lampin M.; Warocquier C.; Legris C.; Degrange M.; Sigot-Luizard M.F. Correlation between substratum roughness and wettability, cell adhesion, and cell migration. *J Biomed Mater Res* 1997, 36, 99–108. 9212394 10.1002/(sici)1097-4636(199707)36:1<99::aid-jbm12>3.0.co;2-e

[6] Schwarz F.; Wieland M.; Schwartz Z.; Zhao G.; Rupp F.; Geis-Gerstorfer J., et al Potential of chemically modified hydrophilic surface characteristics to support tissue integration of titanium dental implants. *J Biomed Mater Res B Appl Biomater* 2009, 88, 544–557. 10.1002/jbm.b.31233 18837448

[7] Gronowicz G.; McCarthy M.B. Response of human osteoblasts to implant materials: Integrin-mediated adhesion. *J Orthop Res* 1996, 14, 878–887. 8982129 10.1002/jor.1100140606

[8] Aronsson B.O.; Lausmaa J.; Kasemo B. Glow discharge plasma treatment for surface cleaning and modification of metallic biomaterials. *J Biomed Mater Res* 1997, 35, 49–73. 9104698 10.1002/(sici)1097-4636(199704)35:1<49::aid-jbm6>3.0.co;2-m

[9] Czarnowska E.; Wierzchon T.; Maranda-Niedbala A.; Karczmarewicz E. Improvement of titanium alloy for biomedical applications by nitriding and carbonitriding processes under glow discharge conditions. *J Mater Sci Mater Med* 2000, 11, 73–81. 15348050 10.1023/a:1008980631780

[10] Duske K.; Wegner K.; Donnert M.; Kunert U.; Podbielski A.; Kreikemeyer B. et al Comparative in vitro study of different atmospheric pressure plasma jets concerning their antimicrobial potential and cellular reaction. *Plasma Processes and Polymers* 2015.

[11] Cools P.; Vanderleyden E.; Dubruel P.; Morent R. Surface analysis of titanium cleaning and activation processes: Non-thermal plasma versus other technique. *Plasma Chemistry Plasma Processing* 2014, 34, 917–932.

[12] Yamamoto H.; Shibata Y.; Miyazaki T. Anode glow discharge plasma treatment of titanium plates facilitates adsorption of extracellular matrix proteins to the plates. *J Dent Res* 2005, 84, 668–671. 15972599 10.1177/154405910508400717

[13] Alves C.M.; Yang Y.; Carnes D.L.; Ong J.L.; Sylvia V.L.; Dean D.D., et al Modulating bone cells response onto starch-based biomaterials by surface plasma treatment and protein adsorption. *Biomaterials* 2007, 28, 307–315. 17011619 10.1016/j.biomaterials.2006.09.010

[14] Singh R.G. Evaluation of the bioactivity of titanium after varied surface treatments using human osteosarcoma osteoblast cells: An in vitro study. *Int J Oral Maxillofac Implants* 2011, 26, 998–1003. 22010082

[15] Durual S.; Pernet F.; Rieder P.; Mekki M.; Cattani-Lorente M.; Wiskott H.W. Titanium nitride oxide coating on rough titanium stimulates the proliferation of human primary osteoblasts. *Clin Oral Implants Res* 2011, 22, 552–559. 10.1111/j.1600-0501.2010.02033.x 21087318

[16] da Silva J.S.; Amico S.C.; Rodrigues A.O.; Barboza C.A.; Alves C. Jr.; Croci A.T. Osteoblastlike cell adhesion on titanium surfaces modified by plasma nitriding. *Int J Oral Maxillofac Implants* 2011, 26, 237–244. 21483875

[17] Huang H.M.; Hsieh S.C.; Teng N.C.; Feng S.W.; Ou K.L.; Chang W.J. Biological surface modification of titanium surfaces using glow discharge plasma. *Med Biol Eng Comput* 2011, 49, 701–706. 10.1007/s11517-011-0742-2 21286829

[18] Chang W.J.; Ou K.L.; Lee S.Y.; Chen J.Y.; Abiko Y.; Lin C.T., et al Type i collagen grafting on titanium surfaces using low-temperature glow discharge. *Dent Mater J* 2008, 27, 340–346. 18717160 10.4012/dmj.27.340

[19] Chang Y.C.; Feng S.W.; Huang H.M.; Teng N.C.; Lin C.T.; Lin H.K., et al Surface analysis of titanium biological modification with glow discharge. *Clin Implant Dent Relat Res* 2013. 10.1111/cid.12141 23981288

[20] Rapuano B.E.; Lee J.J.; MacDonald D.E. Titanium alloy surface oxide modulates the conformation of adsorbed fibronectin to enhance its binding to alpha(5) beta(1) integrins in osteoblasts. *Eur J Oral Sci* 2012, 120, 185–194. 10.1111/j.1600-0722.2012.954.x 22607334 PMC3358816

[21] MacDonald D.E.; Rapuano B.E.; Vyas P.; Lane J.M.; Meyers K.; Wright T. Heat and radiofrequency plasma glow discharge pretreatment of a titanium alloy promote bone formation and osseointegration. *J Cell Biochem* 2013, 114, 2363–2374. 10.1002/jcb.24585 23649564 PMC3786157

[22] Rapuano B.E.; Hackshaw K.; Macdonald D.E. Heat or radiofrequency plasma glow discharge treatment of a titanium alloy stimulates osteoblast gene expression in the mc3t3 osteoprogenitor cell line. *Journal of periodontal & implant science* 2012, 42, 95–104. 22803011 10.5051/jpis.2012.42.3.95 PMC3395001

[23] Rapuano B.E.; Singh H.; Boskey A.L.; Doty S.B.; MacDonald D.E. Heat and radiofrequency plasma glow discharge pretreatment of a titanium alloy: Evidence [corrected] for enhanced osteoinductive properties. *J Cell Biochem* 2013, 114, 1917–1927. 10.1002/jcb.24536 23494951 PMC3810410

[24] Shibata Y.; Hosaka M.; Kawai H.; Miyazaki T. Glow discharge plasma treatment of titanium plates enhances adhesion of osteoblast-like cells to the plates through the integrin-mediated mechanism. *Int J Oral Maxillofac Implants* 2002, 17, 771–777. 12507235

[25] Cho J.; Mosher D.F. Enhancement of thrombogenesis by plasma fibronectin cross-linked to fibrin and assembled in platelet thrombi. *Blood* 2006, 107, 3555–3563. 16391013 10.1182/blood-2005-10-4168 PMC1457097

[26] Li G.; Yang P.; Qin W.; Maitz M.F.; Zhou S.; Huang N. The effect of coimmobilizing heparin and fibronectin on titanium on hemocompatibility and endothelialization. *Biomaterials* 2011, 32, 4691–4703. 10.1016/j.biomaterials.2011.03.025 21463893

[27] Kilkenny C.; Browne W.J.; Cuthill I.C.; Emerson M.; Altman D.G. Improving bioscience research reporting: The arrive guidelines for reporting animal research. *PLoS Biol* 2010, 8, e1000412 10.1371/journal.pbio.1000412 20613859 PMC2893951

[28] Stadelmann W.K.; Digenis A.G.; Tobin G.R. Physiology and healing dynamics of chronic cutaneous wounds. *Am J Surg* 1998, 176, 26S–38S. 9777970 10.1016/s0002-9610(98)00183-4

[29] Sharma C.P.; Hari P.R. Adhesion and stability of blood cells onto polymer substrates: Effect of glow discharge. *J Biomater Appl* 1991, 6, 72–79. 1920070 10.1177/088532829100600105

[30] Kottke-Marchant K.; Anderson J.M.; Umemura Y.; Marchant R.E. Effect of albumin coating on the in vitro blood compatibility of dacron arterial prostheses. *Biomaterials* 1989, 10, 147–155. 2524222 10.1016/0142-9612(89)90017-3

[31] Kawai H.; Shibata Y.; Miyazaki T. Glow discharge plasma pretreatment enhances osteoclast differentiation and survival on titanium plates. *Biomaterials* 2004, 25, 1805–1811. 14738844 10.1016/j.biomaterials.2003.08.032

[32] Clover J.; Gowen M. Are mg-63 and hos te85 human osteosarcoma cell lines representative models of the osteoblastic phenotype? *Bone* 1994, 15, 585–591. 7873286 10.1016/8756-3282(94)90305-0

[33] Feng B.; Weng J.; Yang B.C.; Qu S.X.; Zhang X.D. Characterization of titanium surfaces with calcium and phosphate and osteoblast adhesion. *Biomaterials* 2004, 25, 3421–3428. 15020115 10.1016/j.biomaterials.2003.10.044

[34] Kurachi T.; Nagao H.; Nagura H.; Enomoto S. Effect of a titanium surface on bone marrow-derived osteoblastic cells in vitro. *Arch Oral Biol* 1997, 42, 465–468. 9382711 10.1016/s0003-9969(97)00019-8

[35] Owen T.A.; Aronow M.; Shalhoub V.; Barone L.M.; Wilming L.; Tassinari M.S., et al Progressive development of the rat osteoblast phenotype in vitro: Reciprocal relationships in expression of genes associated with osteoblast proliferation and differentiation during formation of the bone extracellular matrix. *J Cell Physiol* 1990, 143, 420–430. 1694181 10.1002/jcp.1041430304

[36] Kieswetter K.; Schwartz Z.; Hummert T.W.; Cochran D.L.; Simpson J.; Dean D.D., et al Surface roughness modulates the local production of growth factors and cytokines by osteoblast-like mg-63 cells. *J Biomed Mater Res* 1996, 32, 55–63. 8864873 10.1002/(SICI)1097-4636(199609)32:1<55::AID-JBM7>3.0.CO;2-O

