# Supplementary material for: In Vitro Analysis of Fibronectin-Modified Titanium Surfaces
Source: PLoS One. 2016 Jan 5;11(1):e0146219. doi: 10.1371/journal.pone.0146219 (PMC4711664; doi:10.1371/journal.pone.0146219)
Supplement: S1 File — The affidavit of approval from the Animal Care and Ethics Committee of Taipei Medical University, Taipei, Taiwan. (PDF) [file pone.0146219.s001.pdf]

臺北醫學大學實驗動物照護及使用委員會或小組審查同意書

Affidavit of Approval of Animal Use Protocol
Taipei Medical University

動物實驗申請表暨同意書編號 ： LAC-2013-0265

計畫申請人 ： 張維仁　　職稱 ： 副教授

單位 ： 牙醫系　　飼養/應用地點 ： 北醫動物實驗中心

計畫名稱 ： 生物性表面改質之鈦金屬人工牙根之開發研究

本計畫之「動物實驗申請表」業經實驗動物照護及使用委員會
☑ 實質 ☐ 形式 審查通過。

本計畫預定飼養應用之動物如下 ：

動物別/品系	使用數量
紐西蘭大白兔	60

計畫執行期間 ： 從 2014/01/01 到 2015/12/31

The animal use protocol listed below has been reviewed and approved by the Institutional Animal Care and Use Committee or Panel (IACUC/ IACUP)

Protocol Title : Development of biological surface modified tianium dental implant

IACUC Approval No : LAC-2013-0265

Period of Protocol : Valid From : 2014/01/01 To : 2015/12/31

Principle Investigator (PI) : Wei-jen, Chang

實驗動物照護及使用委員會召集人

IACUC Chairman : [signature] 蕭哲志

Date : 2013/12/30

臺北醫學大學動物實驗倫理 3R 說明文件

計畫申請人： 張維仁 職 稱： 副教授

單 位 ： 牙醫系

計畫名稱： 生物性表面改質之鈦金屬人工牙根之開發研究

1. 本實驗動物操作部份是否有其他替代方案？

■ 無法替代，說明：以觀察動物接受手術後傷口癒合才能得知骨整合之過程

□ 有替代方案，但仍須進行本動物實驗，理由如下：

2. 是否已儘量減少動物使用？

■ 已減量，說明：原實驗設計動物使用量 120 隻，重新設計與計算後，可減量、精緻化，預估使用 60 隻即可達成實驗目標。

□ 無法減量，理由如下：□相關法規或技術文件規定：

□其他

3. 操作人員是否已受過專業技術訓練，以避免無謂的動物傷害？

■ 是，受訓經歷： 學校專業課程

□ 無，培訓計畫如下：□農委會專業課程 □本校專業課程 □其他

4. 實驗動物飼養環境為何？

■ 於本校實驗動物中心飼養，由專業人員集中管理，飼養環境符合規範。

□ 於校外合格單位飼養，單位：

□ 不飼養，操作後立即結束實驗。

5. 本實驗進行實驗動物基因改造之理由？

■ 未進行實驗動物基因改造。

□ 經查詢國家動物中心實驗鼠種源庫，無符合需求之基因改造動物。

□ 相關基因改造動物難以取得或過於昂貴，必須自行開發。

6. 本實驗基因改造動物於開發完成後如何進行保種與分享？

■ 未進行實驗動物基因改造。

□ 使用國家動物中心實驗鼠種源庫服務。

□ 其他種源管理單位：

□ 不保種或不分享，理由：

7. 研究成果是否有助形成其他實驗之動物減替代方案？

□ 難以評估。

■ 有明確幫助，說明如下：可減少以大型動物進行的實驗，如：迷你豬

茲保證以上填寫事項皆正確無誤，並遵守動物倫理 3R「取代(Replace)」、「減量(Reduce)」、及實驗「精緻化(Refine)」動物實驗研究之基本原則。

計劃申請人簽名：[illegible]
